# Supplementary material for: Voltage detected single spin dynamics in diamond at ambient conditions
Source: Nat Commun. 2025 Apr 14;16:3518. doi: 10.1038/s41467-025-58635-3 (PMC11997230; doi:10.1038/s41467-025-58635-3)
Supplement: Supplementary file 1 — Supplementary Information [file 41467_2025_58635_MOESM1_ESM.pdf]

Supplementary Information for

Voltage detected single spin dynamics in diamond at ambient conditions

Sergei Trofimov[1], Klaus Lips[1], and Boris Naydenov[1,*]

[1]Berlin Joint EPR Laboratory and Department Spins in Energy Conversion and Quantum Information Science (ASPIN), Helmholtz-Zentrum Berlin für Materialien und Energie, Hahn-Meitner-Platz 1, 14109 Berlin, Germany
[*]Corresponding author. Email: boris.naydenov@helmholtz-berlin.de

Contents

1 Materials and Methods 2

2 NV centre imaging 3

3 Model for the generation of the photovoltage (PV) 6

4 PV signal as a function of the V_{AC} and the tip-electrode distance 7

5 PV detection of magnetic resonance and Rabi oscillations 9

6 References 12

Supplementary Note 1: Materials and Methods

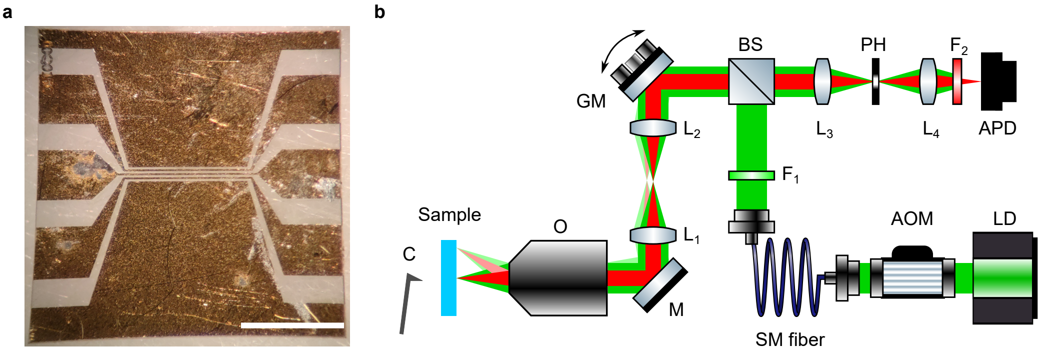

Supplementary Figure 1: Sample and setup. **a**, Diamond plate with metal electrodes. The scale bar is 1 mm. **b**, Schematics of the experimental setup. The laser (green) and the detected PL (red) beam paths can be controlled by the galvo mirrors (GM). The beam paths when the mirrors are rotated are shown in light green and light red, correspondingly. Letter abbreviations: C – cantilever, O – objective, L – lens, GM – galvo mirrors, BS – beam splitter, PH – pinhole, F – optical filter, APD – Avalanche photodiode, SM – single mode, AOM – acousto-optic modulator, LD – laser diode. Figure adapted from [4].

Supplementary Note 2: NV centre imaging

Contact potential difference (CPD) and photoluminescence (PL) images were obtained by scanning the laser around the cantilever and correlating its position with the measured surface potential and luminescence, correspondingly (see Supplementary Fig. 2a, b). Photovoltage (PV) images were derived from CPD images by subtracting from the latter the CPD signal in the dark. The PV images were then used to obtain PV_{NV}, PV_{surf}, and contrast images to remove the influence from the diamond surface (see Supplementary Fig. 2c–e). For this, a PV signal from each of single NV centres was fitted with a sum of a two-dimensional (2D) Gaussian function $PV_{NV}(x,y) = Se^{-\frac{x^2+y^2}{2\sigma^2}}$ and a 2D surface $PV_{surf}(x,y) = Ax + By + C$ representing the background. The PV_{surf} was chosen to be a plane to account for a change in the PV signal with the distance to the cantilever. The imaging contrast was calculated as:

$$C_{PV}^{im}(x,y) = \frac{PV_{NV}(x,y)}{|PV_{NV}(x,y)| + |PV_{surf}(x,y)|} \quad (1)$$

In this equation we use absolute values in the denominator, since the PV_{NV} and PV_{surf} signals have different signs.

As pointed out in the main text, at high scanning rate PV images seem to lag behind PL images (the fast scan direction is from left to right) due to the signal response time (on the order of 10 ms). For decreased scanning rate, however, this effect is mitigated, and the positions of an NV in the PL and the PV images are overlapping within the diffraction limit. An example of this effect is shown in Supplementary Fig. 2a, b, where the difference in the PL and the PV positions of the NV was calculated to be 69 ± 1 nm (scanning rate 1 µm/s).

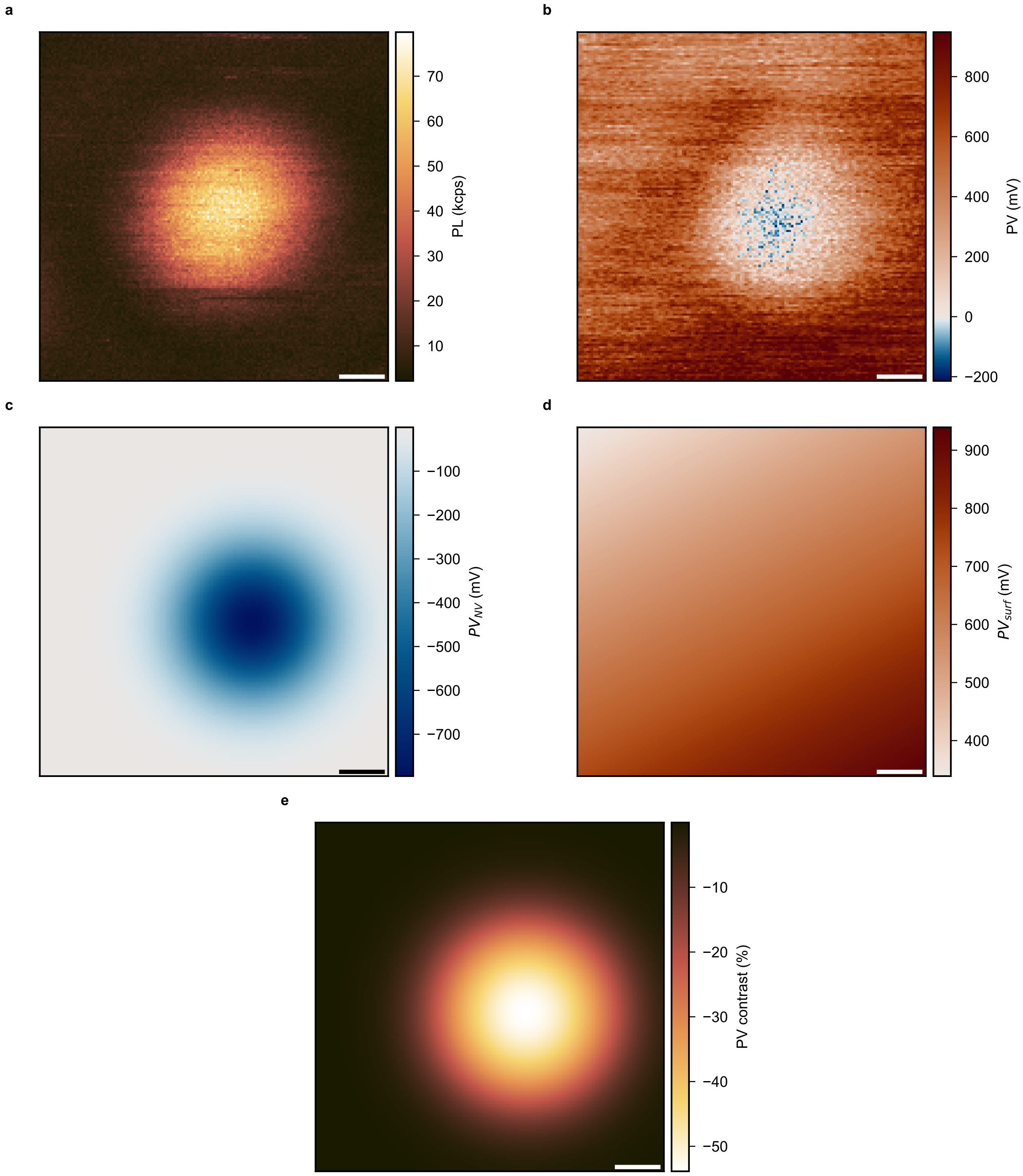

Supplementary Figure 2: NV centre imaging. **a**, PL image of a single NV centre **b**, PV image obtained simultaneously with (**a**). The colour scale is chosen so that positive PV is brown, negative – blue and the PV equal to zero is white. **c**, **d** PV_{NV} and PV_{surf} images obtained from fitting (**b**). **e**, PV contrast image obtained from fitting (**b**). The scale bars are 100 nm.

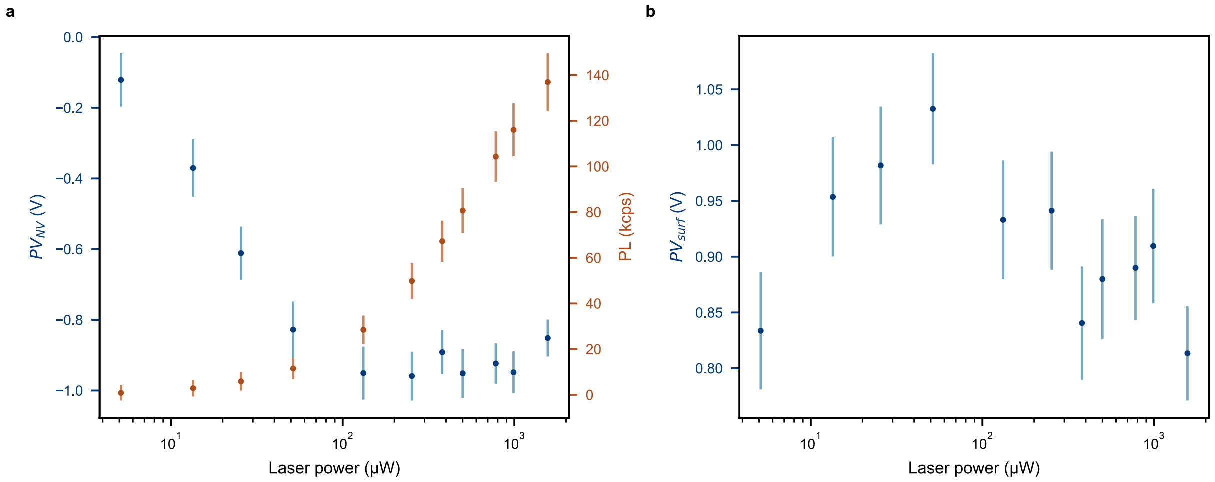

Supplementary Figure 3: Saturation curves. **a**, PV and PL saturation curves from a single NV centre (PV and PL signals from surface are subtracted). The PV_{NV} signal is expected to be quadratic in the laser power due to the two-photon excitation processes, but instead we observe a saturation behaviour. **b**, PV saturation curve from surface. Each data point represents the mean value of the signal recorded over 60 seconds with a 1 ms sampling rate ($6 \cdot 10^4$ points). Error bars indicate the standard deviation (SD) of the signal during this period.

Supplementary Note 3: Model for the generation of the photovoltage (PV)

As explained in the main text, the model for the generation of PV is based on surface acceptor states able to trap the free charge carriers that are attracted to the cantilever by the oscillating AC potential. Using the AC voltage amplitude V_{AC} and the carrier mobility (μ_h or μ_e for holes and electrons, respectively) for diamond, one can calculate the time t needed for carriers to drift by the distance between the cantilever and the grounding contact l, using the formula $t_{e,h} = \frac{l^2}{\mu_{e,h} V_{AC}}$. Note that here the voltage was assumed to act like direct current (DC), which is justified by the following calculation. We also do not consider here the potential drop between the tip and the sample surface. For the typical experimental parameters $V_{AC} \sim 1$ V, $l \sim 1$ µm, μ_h and $\mu_e \sim 1000$ cm^{2}V^{-1}s^{-1} (see, for example, [1]), this time is $t \approx 100$ ps, which is 10^7 times shorter than the typical voltage oscillation period (1 ms for a frequency $f_{AC} \sim 1$ kHz).

Considering just the surface states, one can assume a simple model, where during the half of the oscillation period of V_{AC} excited holes gather below the cantilever, populating the surface states, and in the next half of the period they are released from these trap states. The photovoltage signal, measured by the cantilever, is averaged over several periods and is negative, when the laser is focused on the tip (Fig. 4a) and positive, when the illuminated spot is several microns away from the cantilever (Fig. 4b). When the laser is focused on an NV centre, additional holes and electrons are emitted, contributing to the charge trapped under the tip. Since at a laser power above ~ 20 µW the PV signal from the surface stays in the range of 0.8–1.05 V not showing a strong dependence on the laser power (see Supplementary Fig. 3b), we assume, that at these laser powers the AC potential applied to the cantilever is screened by the already captured holes. Therefore, the additional holes from an NV centre cannot be trapped under the cantilever. The electrons, however, can easily be captured by the surface acceptors. This leads to a decrease in the average charge trapped under the tip and hence to a drop in the monitored photovoltage (Fig. 4c).

Supplementary Note 4: PV signal as a function of the V_{AC} and the tip-electrode distance

Our qualitative model for the origin of the PV signal suggests that the applied for KPFM experiment V_{AC} controls the photocarrier motion. This is supported by additional measurements where the V_{AC} and the distance between the cantilever and the grounding electrode were altered. In the first set of experiments, we changed the AC voltage amplitude and monitored how it affected the PV image of NV centres. In the second set, the V_{AC} was kept constant, while the distance between the cantilever and the grounding contact was increased. The results are shown in Supplementary Fig. 4. In both cases the image contrast changes its sign (the NV centres show positive signal on the negative signal from the surface) due to the decrease in the electric field and consequent change of the carrier motion from drift to diffusion, which leads to a higher noise and a lower absolute contrast. The negative PV from the surface at long tip-electrode distances is attributed to the fact, that even when the laser is focused far away from the cantilever, the tip is still illuminated by the scattered light of a low intensity, which is enough to excite holes from the surface states trapping the negative charge under the cantilever. Thus, when NV centres are excited, additional holes from them diffuse and eventually reach the cantilever, populating the surface states and decreasing the trapped negative charge. The electrons from NV centres in this case are weakly attracted to the tip due to the Coulomb repulsion.

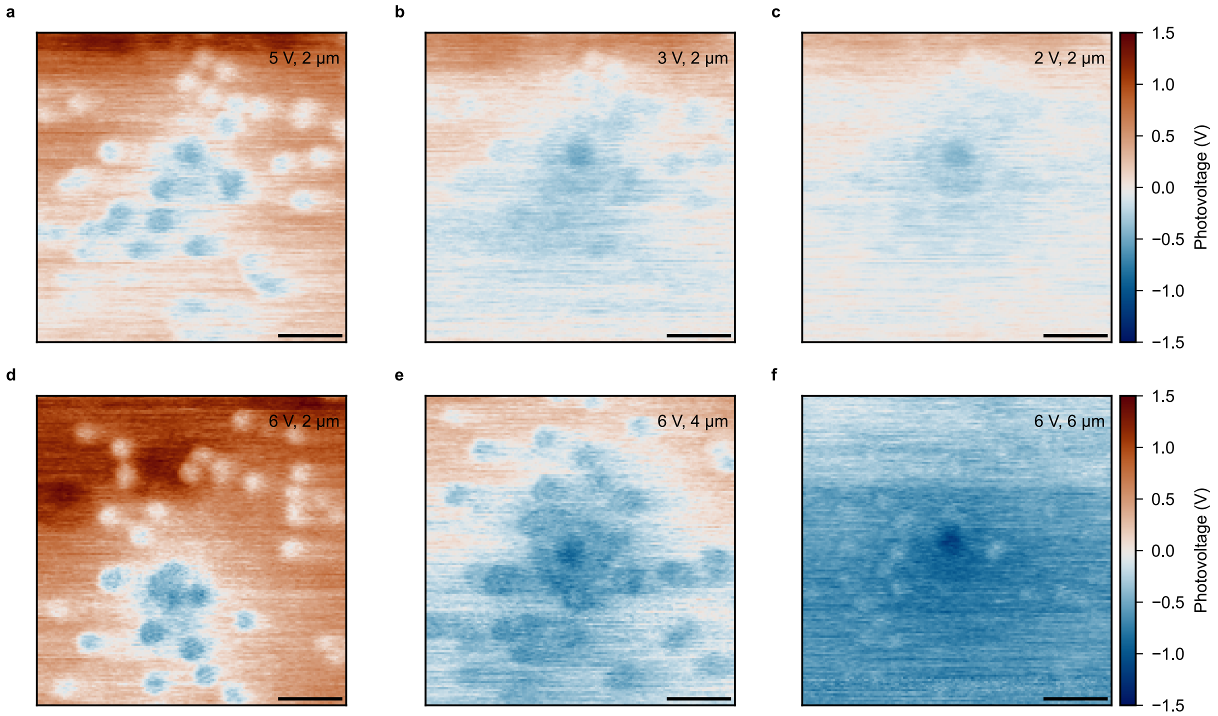

Supplementary Figure 4: PV images of NV centres obtained at different AC potential amplitudes V_{AC} and at different distances between the cantilever and the grounding electrode (given in top right for every scan). **a**, **b**, **c**, The distance between the cantilever and the grounding contact is constant and the V_{AC} amplitude is changed. **d**, **e**, **f**, The V_{AC} amplitude is constant and the distance between the cantilever and the grounding contact is changed. The scale bars are 1 μm.

Supplementary Note 5: PV detection of magnetic resonance and Rabi oscillations

As pointed out in the main text, the PV signal has a settling time on the order of 10 ms. For magnetic resonance experiments in a CW mode, it means that the acquisition time per MW frequency should be long enough to take into account changes in the signal. However, even with 1 s waiting time the line position in a Surface Voltage Detected Magnetic Resonance (SVDMR) spectrum appears shifted to higher frequencies (compared to the ODMR line, see Supplementary Fig. 5a). Moreover, due to the long integration time in this case, slow changes of the signal coming from the acoustic noise and vibrations picked up by the cantilever increase the standard deviation for each measurement point. To resolve these issues, we perform the SVDMR experiments in a pulsed manner, where MW output is modulated by a low-frequency envelope as shown in Supplementary Fig. 5c. This is done by an MW switch (ZASWA-2-50DRA+, Mini-Circuits) that transmits the MWs to the sample depending on a logic pulse (envelope). For each MW frequency, the pulse block was repeated N times and averaged to obtain the signal demonstrated in Supplementary Fig. 5d. The analysis was performed in the following way. From this time trace, the "signal" ($PV^{on}(\nu)$) and "reference" (PV^{off}) parts are derived averaging the PV in the corresponding windows to obtain the $\Delta PV^{MR} = PV^{on}(\nu) - PV^{off}$ value, which is plotted in SVDMR figures. Using that $PV_{NV} = PV - PV_{surf}$, the fact that the PV_{surf} does not depend on the MW application, and that PV_{NV}^{off} is simply PV_{NV} obtained on the measured NV centre, the magnetic resonance contrast defined in Eq. 2 of the main text can be rewritten in the following way:

$$C_{PV}^{MR} = \frac{PV^{on}(\nu) - PV_{surf}^{on}(\nu) - PV^{off} + PV_{surf}^{off}}{PV_{NV}^{off}} = \frac{PV^{on}(\nu) - PV^{off}}{PV_{NV}} = \frac{\Delta PV^{MR}(\nu)}{PV_{NV}}$$

For comparison, the SVDMR obtained in pulsed mode with the same integration time per MW frequency point and the same number of accumulations as the one in Supplementary Fig. 5a is shown in Supplementary Fig. 5b.

To be able to compare signal amplitudes in both modes, in Supplementary Fig. 5a we have plotted $\Delta\widetilde{PV}^{MR} = PV(\nu) - PV(\nu_{off\_res})$ and $\Delta\widetilde{PL}^{MR} = PL(\nu) - PL(\nu_{off\_res})$, where $PV(\nu_{off\_res})$ ($PL(\nu_{off\_res})$) is the PV (PL) signal at a non-resonant (off-resonant) frequency. In our case, we have used an average of the PV and PL signals at the first two frequency points (2.85 and 2.852 GHz) for estimation of these values.

The Rabi oscillations assume application of short MW pulses (on the order of 10–100 ns) to coherently manipulate the spin of interest. Thus, for PV detected Rabi oscillations, we used a similar envelope pulse sequence with envelope modulation of MW [2] (see Supplementary Fig. 5e and f). The analysis is analogous to the one described above for SVDMR. Now $\Delta PV^{Rabi} = PV^{on}(\tau) - PV^{off}$, where PV^{on} is obtained with MWs applied for time τ at the resonance frequency ν_{res}. The contrast of the signal is

$$C_{PV}^{Rabi} = \frac{PV^{on}(\tau) - PV^{off}}{PV_{NV}} = \frac{\Delta PV^{Rabi}(\tau)}{PV_{NV}} \quad (2)$$

According to our observations, an increase in the laser power can result in a reduction of the signal settling time, mentioned above and clearly visible in Supplementary Fig. 5d. Therefore, higher powers can be used to reduce the pulse block length (see Supplementary

Fig. 5c, e), decreasing the total accumulation time. However, high laser power can also result in a decrease of the contrast, when the optical excitation rate becomes comparable to the Rabi frequency. Thus, the laser power was chosen to be high enough to reduce the length of the pulse block, but still have the maximum contrast, which we confirmed with conventional optically detected Rabi oscillations. For typical measurements, the laser power of 500 µW was used for SVDMR experiments, and 750 µW for PV-detected Rabi oscillations. The laser pulse length for Rabi experiments was chosen to be 1 µs, so that it is long enough to polarize the NV centre in the $m_s = 0$ state and short enough not to lose the spin contrast [3].

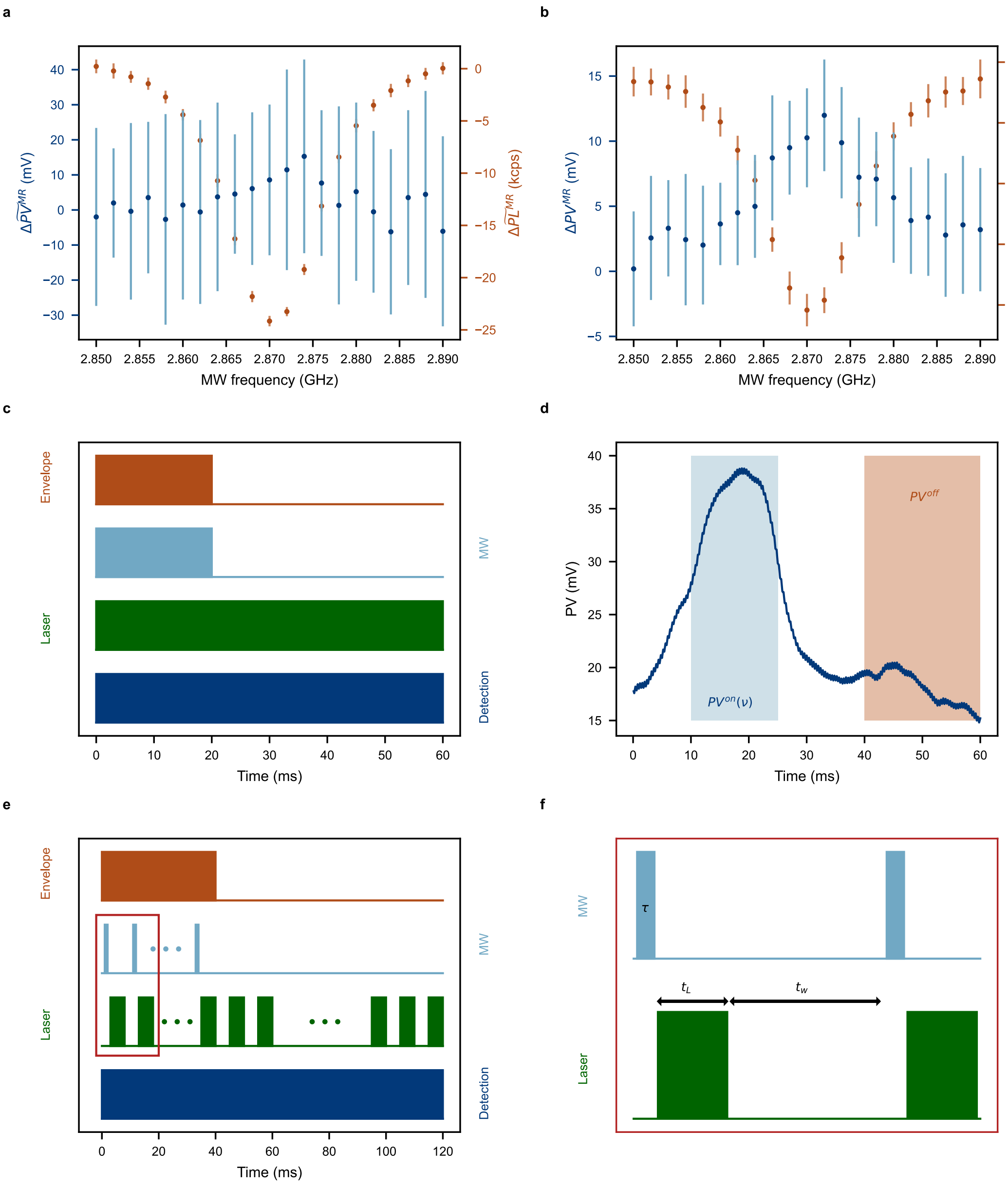

Supplementary Figure 5: NV centre spin state detection. **a**, SVDMR and ODMR in CW mode (1 s per point, 31 accumulations) from a single NV centre **b**, SVDMR and ODMR in the pulsed mode (1 s per point, 31 accumulations) on the same NV as (**a**). Error bars represent the SD of 31 independent measurements (accumulations). **c**, Pulse block for SVDMR experiments. The envelope (and, hence, MW) pulse length is 20 ms, while the total block length is 60 ms. At each MW frequency this block is repeated N times (typically 100). **d**, The obtained time trace of the PV signal after averaging over N pulse block repetitions with resonant MW. The light blue and light brown regions show the "signal" and the "reference" windows correspondingly. **e**, Pulse block for Rabi experiments. The envelope pulse length is 40 ms, while the total block length is 120 ms. For each MW pulse length τ this block is repeated N times (usually 100). **f**, Zoomed-in part from (**e**), showing the Rabi sequence. The laser pulse length t_L was set to be 1 µs and the waiting time t_w was set to be 1.5 µs, while the MW pulse length τ was varied during the experiment.

Supplementary References

[1] C. E. Nebel, "Electronic properties of CVD diamond," *Semiconductor Science and Technology*, vol. 18, pp. S1–S11, Mar. 2003. Number: 3.

[2] M. Gulka, E. Bourgeois, J. Hruby, P. Siyushev, G. Wachter, F. Aumayr, P. R. Hemmer, A. Gali, F. Jelezko, M. Trupke, and M. Nesladek, "Pulsed Photoelectric Coherent Manipulation and Detection of N-V Center Spins in Diamond," *Physical Review Applied*, vol. 7, p. 044032, Apr. 2017.

[3] M. Gulka, D. Wirtitsch, V. Ivády, J. Vodnik, J. Hruby, G. Magchiels, E. Bourgeois, A. Gali, M. Trupke, and M. Nesladek, "Room-temperature control and electrical readout of individual nitrogen-vacancy nuclear spins," *Nature Communications*, vol. 12, p. 4421, July 2021.

[4] S. Trofimov and B. Naydenov "Combined Confocal-Atomic-Force Microscope Setup for Quantum Sensing Applications with Sub-diffractional Spatial Resolution.," *physica status solidi (a)*, vol. 222, p. 2400541, 2025
